# Supplementary material for: High Performance GaN-Based Ultraviolet Photodetector via Te/Metal Electrodes
Source: Materials (Basel). 2023 Jun 24;16(13):4569. doi: 10.3390/ma16134569 (PMC10342670; doi:10.3390/ma16134569)
Supplement: Supplementary file 1 [file materials-16-04569-s001.zip › materials-2429345-supplementary.pdf]

materials

MDPI

*Supplementary material*

# High Performance GaN-Based Ultraviolet Photodetector via Te/Metal Electrodes

**Sheng Lin [1], Tingjun Lin [2], Wenliang Wang [2,*], Chao Liu [3] and Yao Ding [1,*]**

[1] School of Materials Science and Engineering, Wuhan University of Technology, Wuhan 430070, China; 303494@whut.edu.cn
[2] Department of Electronic Materials, School of Materials Science and Engineering, South China University of Technology, Guangzhou 510640, China; 202120121552@mail.scut.edu.cn
[3] State Key Laboratory of Crystal Materials, School of Microelectronics, Institute of Novel Semiconductors, Shandong Technology Center of Nanodevices and Integration, Shandong University, Jinan 250100, China; chao.liu@sdu.edu.cn
* Correspondence: wenliangwang@scut.edu.cn (W.W.); ydingaf@whut.edu.cn (Y.D.)

Figure S1 shows the X-ray diffraction patterns of Te nanowire prepared by hydrothermal synthesis, from which we can see that the Te peaks match perfectly with the standard plots, indicating that the tellurium nanowires we obtained are of very high quality.

Figure S2 shows Electronic structures of Te/GaN heterojunction. Based on the result, it shows that due to the narrow bandgap and the semimetal property of Te, the whole junction has the metallic property at the interface, which proves that by contacted with Te, the mobility of the photogenerated carriers in Te/GaN can be greatly improved.

The response speed of a PD is usually characterized by the rise time (τr) and the fall time (τf ). The rise time is defined as the time interval for the response to rise from 10% to 90% of its peak value, whereas the fall time is defined as the time interval for the response to decay from 90% to 10% of its peak value. From the time-resolved optical response curve shown in Figure S3, it can be calculated that the response and decay time of the Te/GaN PD are 100ms and 270ms, respectively. In the rising phase, the maximum current value is 31.3615mA, the slope of the fitting function is 240.95, and the fitting coefficient is 0.847. In the falling phase, the slope of the fitting function is -92.93, and the fitting coefficient is 0.76.

Figure S4 shows shows the light response curve of GaN at 254nm and 365nm. It can be seen from the figure that GaN has similar light response behavior to Te/GaN heterojunction, that is, it has better light response at 365nm.

Figure S5 shows optical images of other photoelectric devices we have tested. After many tests, we got nearly consistent response results and presented them in the manuscript.

Figure S6 shows I-V curves of Te-enhanced GaN-based PDs displayed in Figure S5.

Figure S7 shows our Te nanosheet /GaN device. In order to eliminate the adverse consequences that may be caused by the same gold electrode contacting Te and GaN at the same time, we deposited Au/Ti electrodes (the area shown in the dotted line box in the figure) on the Te nanosheet alone. After a series of optical response tests, the results obtained are almost consistent with the Te nanowires /GaN devices.

**The computation of responsivity (R) and detectivity (D*)**: Here, the responsivity and detectivity are defined by following equations:

$$\mathrm{R} = \frac{\mathrm{I_{ph}} - \mathrm{I_d}}{\mathrm{PS}} \quad (1)$$

$$\mathrm{D}^* = \frac{\mathrm{A}^{1/2}\mathrm{R}}{\sqrt{2\mathrm{qI}_d}} \quad (2)$$

where $I_{ph}$, $I_d$, P, S, A and q are the photocurrent, dark current, incident optical power density, light absorption area of the photodetector, effective device area and fundamental

unit charge, respectively.Here, $I_d$, S, A and q equal to $3.1\times10^{-7}$ A ,1 $cm^2$, $3.604\times10^{-5}$ $cm^{-2}$, and $1.60\times10^{-19}$ C, respectively. $I_{ph}$ is the response current under different intensities of light. P ranges from 8.49 to 120.6 $mW/cm^2$. When calculating the maximum value of R and $D^*$, we take the photocurrent value is 42 mA when the light intensity is 8.49 $mW/cm^2$ and the bias voltage is -10V. According to the above formula, the maximum values of R and $D^*$ are 4951 mA/W and $1.79\times10^{14}$ Jones respectively.

**The computation of external quantum efficiency (EQE):** the EQE as the power density varies by using the following equation:

$$EQE = \frac{Rhc}{e\lambda} \tag{3}$$

where h is Planck constant, c is the speed of light, e is the electronic charge and $\lambda$ is the wavelength of the incident light.Here, $h$, c, e and λ equal to $6.63\times10^{-34}$ J·s, $3\times10^{8}$ m/s, $1.60\times10^{-19}$ C，and 365 nm. When calculating the maximum value of EQE, we take the R value is 4951 mA/W. And we calculate that the maximum value of EQE is 169%.

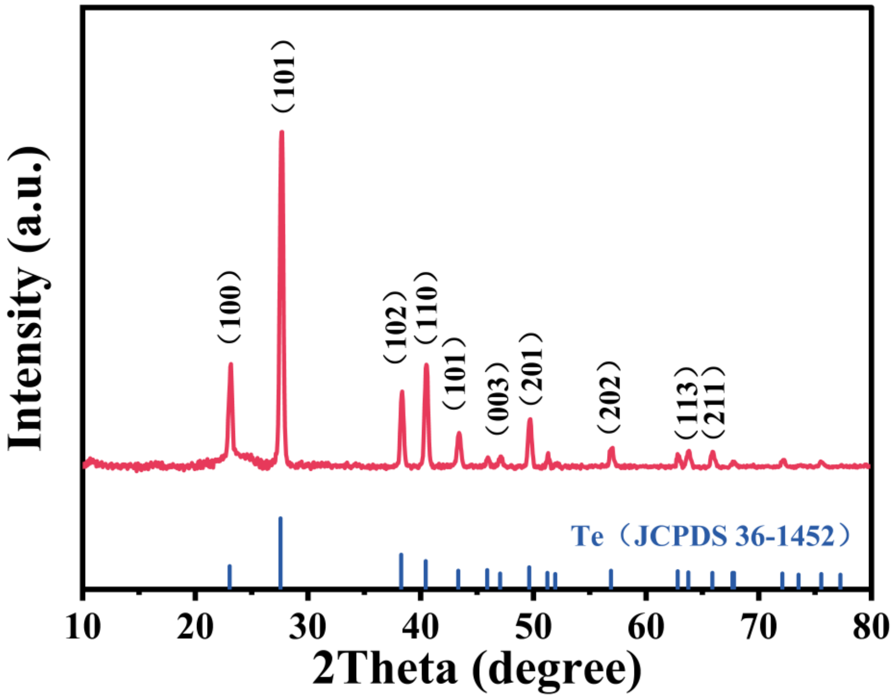


**Figure S1.** X-ray diffraction patterns of Te nanowire.

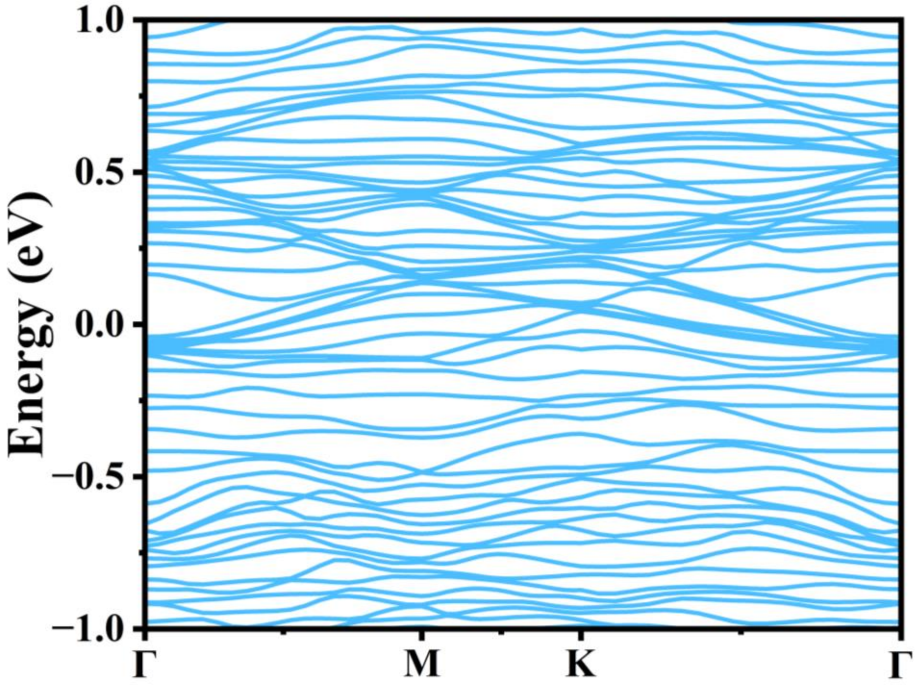


**Figure S2.** Electronic structures of Te/GaN heterojunction.

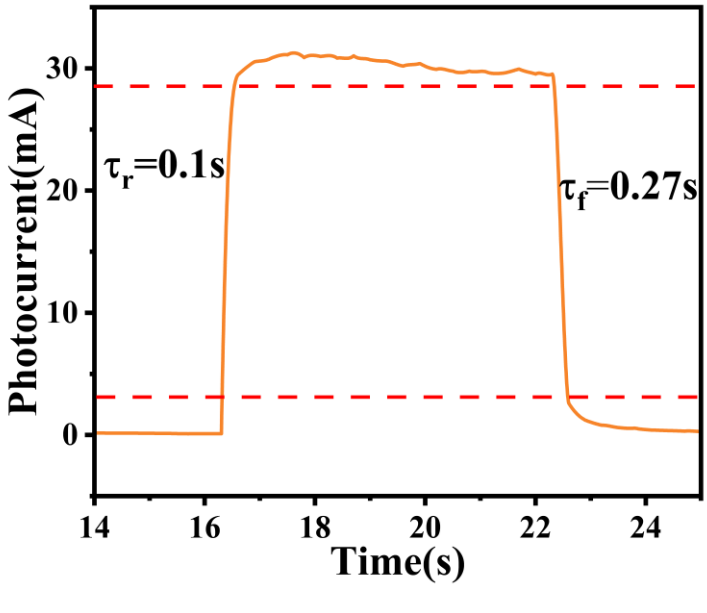


**Figure S3.** The rise time and the fall time of Te/GaN Heterojunction at 1V under 365 nm light illumination.

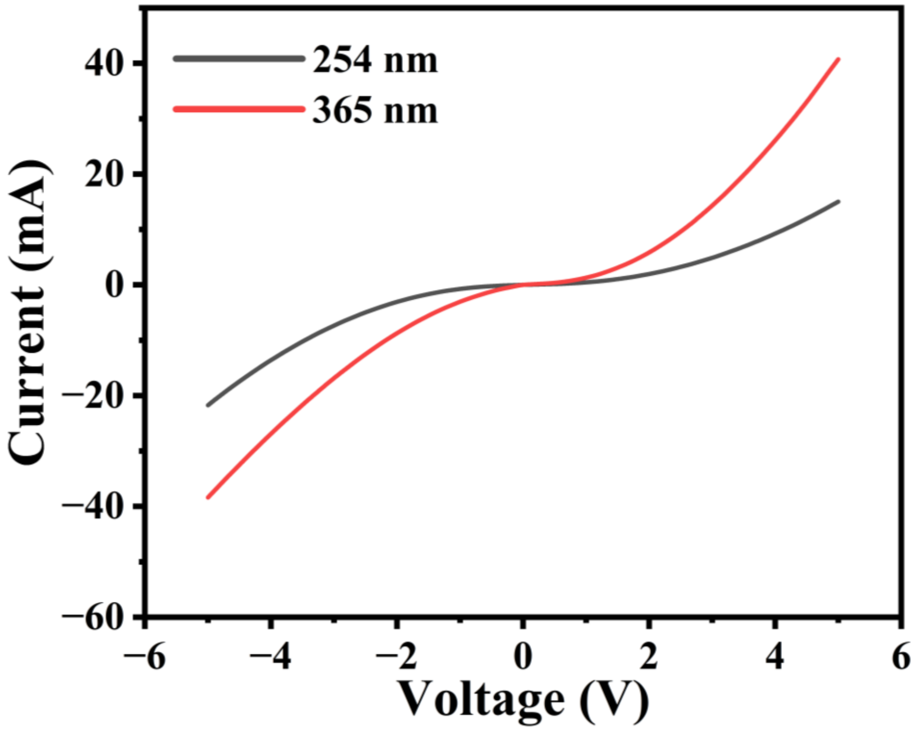


**Figure S4.** Photovoltaic behavior of GaN at 254 nm and 365 nm.

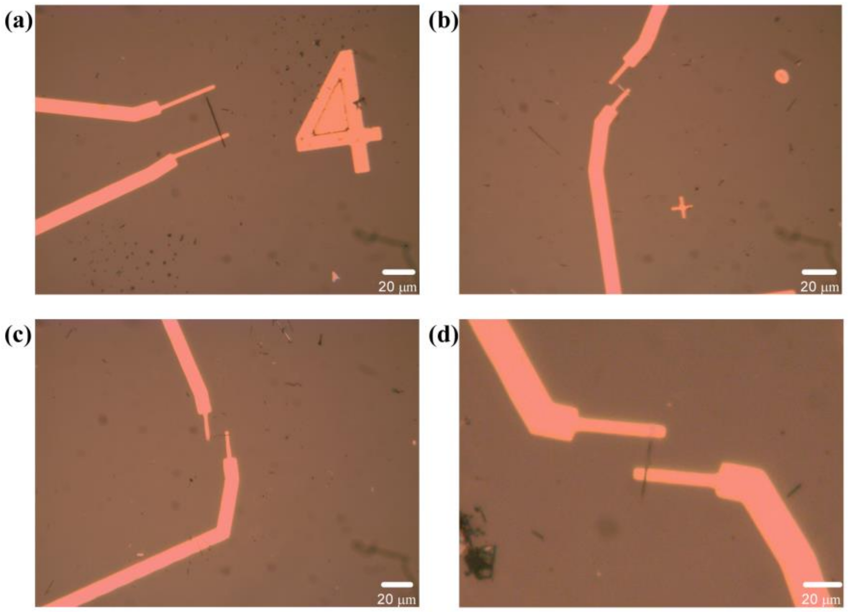


**Figure S5.** (**a**–**d**) Devices of Te-enhanced GaN-based PDs.

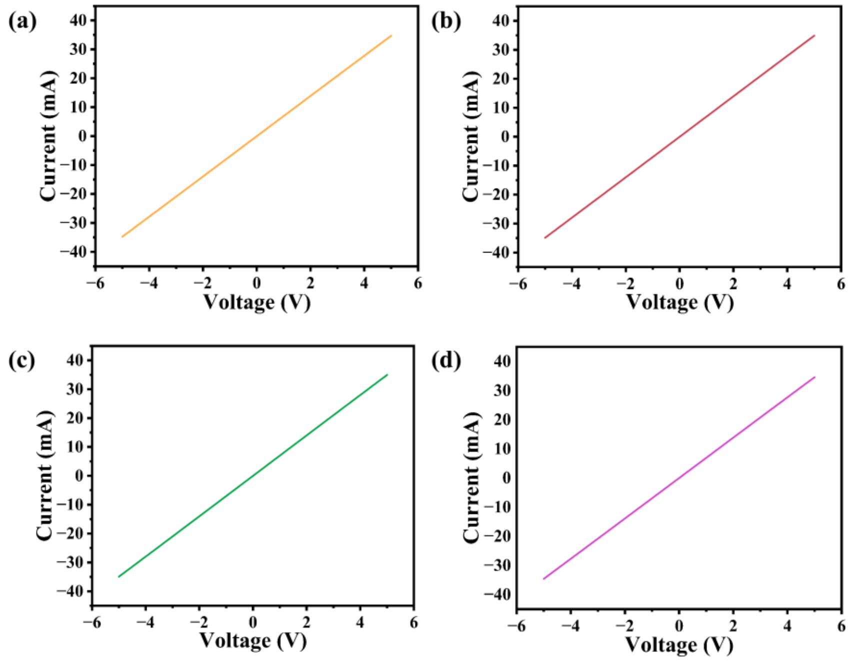


**Figure S6.** I–V curves of Te-enhanced GaN-based PDs shown in Figure S5 (**a**–**d**).

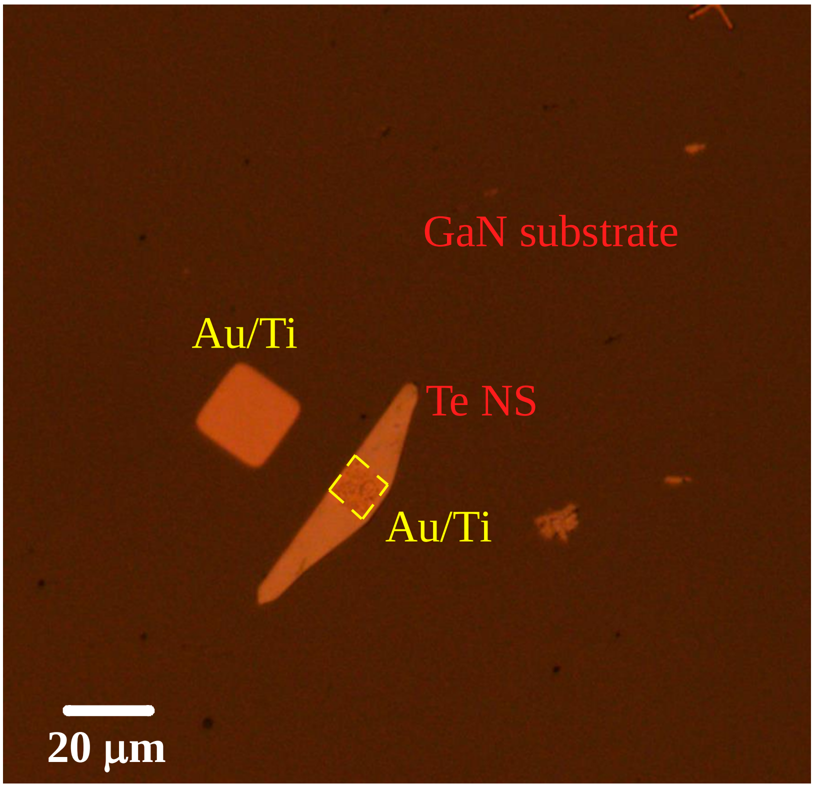


**Figure S7.** Devices of Te nanoplates/GaN heterojunction.
